# Acute Respiratory Distress Syndrome: Pathophysiological Insights, Subphenotypes, and Clinical Implications—A Comprehensive Review

**DOI:** 10.3390/jcm14155184

**Published:** 2025-07-22

**Authors:** Mairi Ziaka, Aristomenis Exadaktylos

**Affiliations:** Department of Emergency Medicine, Inselspital, Bern University Hospital, University of Bern, 3010 Bern, Switzerland; aristomenis.exadaktylos@insel.ch

**Keywords:** acute respiratory distress syndrome, alveolo-capillary barrier dysfunction, biomarkers, inflammatory cascades, lung injury, precision medicine, subphenotypes

## Abstract

Increased epithelial and endothelial permeability, along with dysregulated inflammatory responses, are key aspects of acute respiratory distress syndrome (ARDS) pathophysiology, which not only impact the lungs but also contribute to detrimental organ crosstalk with distant organs, ultimately leading to multiple organ dysfunction syndrome (MODS)—the primary cause of morbidity and mortality in patients with lung injury (LI) and ARDS. It is predominantly manifested by hypoxemic respiratory failure and bilateral pulmonary infiltrates, which cannot be fully attributed to cardiac failure or hypervolemia, but rather to alveolo-capillary barrier dysfunction, dysregulated systemic and pulmonary inflammation, immune system abnormalities, and mechanical stimuli-related responses. However, these pathological features are not uniform among patients with ARDS, as distinct subphenotypes with unique biological, clinical, physiological, and radiographic characteristics have been increasingly recognized in recent decades. The severity of ARDS, clinical outcomes, mortality, and efficacy of applied therapeutic measures appear significant depending on the respective phenotype. Acknowledging the heterogeneity of ARDS and defining distinct subphenotypes could significantly modify therapeutic strategies, enabling more precise and targeted treatments. To address these issues, a comprehensive literature search was conducted in PubMed using predefined keywords related to ARDS pathophysiology, subphenotypes, and personalized therapeutic approaches. Optimizing the identification and characterization of discrete ARDS subphenotypes—based on clinical, biological, physiological, and radiographic criteria—will deepen our understanding of ARDS pathophysiology, promote targeted recruitment in prospective clinical studies to define patient clusters with heterogeneous therapeutic responses, and support the shift toward individualized treatment strategies.

## 1. Introduction

ARDS is a clinical syndrome characterized by the sudden onset of hypoxemic respiratory failure and bilateral infiltrates on chest imaging, which cannot be fully attributed to cardiac failure or hypervolemia [1] and is characterized by inflammatory LI with a mortality rate near 40% [2]. A wide range of etiologies can cause ARDS through either a direct (pulmonary) or indirect (extrapulmonary) mechanism. Common indirect causes include sepsis, non-thoracic major trauma, transfusion of blood products, and pancreatitis, whereas direct causes include pneumonia (bacterial, fungal, or viral), aspiration of gastric contents, and pulmonary or inhalational trauma [3,4]. Recently, based on recent developments in ARDS research, a new global definition of the syndrome was proposed by 32 intensive care ARDS experts. This new definition incorporates the use of high-flow nasal oxygen with a minimum flow rate of ≥30 L/min, allows for the assessment of hypoxemic respiratory failure using pulse oximetry (oxygen saturation to inspired oxygen fraction ratio, SpO_2_/FiO_2_  ≤  315) in addition to arterial blood gases, and includes ultrasound modalities to identify bilateral opacities, particularly for use in resource-limited settings [5]. In patients with ARDS, pulmonary vascular permeability and lung weight increase, along with a reduction in aerated lung tissue, due to the acute inflammatory response that injures the microvascular endothelium and alveolar epithelium of the alveolar-capillary barrier [1]. Epidemiological studies indicate that ARDS is a prevalent condition, with over three million individuals diagnosed worldwide each year, representing approximately 10% of intensive care unit (ICU) admissions [6].

The disease process progresses through three overlapping phases: the acute exudative/inflammatory phase, the proliferative phase, and the fibrotic phase [7]. Indeed, ARDS is characterized by dysregulated inflammatory mechanisms, including the activation of the immune system, the formation of danger-associated molecular patterns (DAMPs), the activation of the innate immune response, the generation of neutrophil extracellular traps and histone release, and the production of reactive oxygen species, leukocyte proteases, chemokines, and cytokines, and uncontrolled activation of coagulation pathways [8,9,10,11]. In addition, alveolar macrophages release cytokines, including interleukins (IL) 1, 6, 8, and 10, as well as tumor necrosis factor α (TNF-α), which stimulate neutrophils to release proinflammatory molecules and promote extracellular matrix production by fibroblasts [12,13]. Fibroblast activation is a central process in the pathophysiology of ARDS and includes proliferation, particularly of interstitial fibroblasts, migration into the fibrinous exudate within the alveolar airspace, replication, and secretion of extracellular matrix proteins, including collagen. These processes impact alveolar clearance and functional recovery, potentially leading to interstitial and intra-alveolar fibrosis [14,15,16].

Almost 60 years after the first description of ARDS by Ashbaugh and colleagues [17], effective therapeutic options remain limited and are primarily supportive, contributing to the persistently high mortality associated with the syndrome [18,19,20]. A potential explanation is the significant heterogeneity of the disease, including physiological and clinical factors, etiology, time of onset, the presence of inflammation and associated biomarkers, and genetic differences [21,22]. Consequently, this has led to the proposal of dividing ARDS into distinct groups based on specific and consistent observable characteristics, also referred to as subphenotypes [21], which could unmask the clinical and biological heterogeneity that hinders the development of new therapeutic strategies. This approach could also help evaluate “unsuccessful” studies with disappointing results regarding therapeutic implications, as these strategies may be beneficial for one subphenotype and detrimental for another [23]. However, as highlighted by the LIVE trial, which randomized patients with moderate-to-severe ARDS to receive either standard ventilatory therapy or personalized therapy based on their lung morphology, the personalization of ventilator strategy did not lead to improved outcomes, possibly due to the misclassification of 21% of patients. Thus, classification accuracy remains an issue of critical importance [24]. Indeed, although our understanding of ARDS pathophysiology and the heterogeneity of the syndrome has advanced significantly, several key challenges remain. These include the establishment of an optimal and standardized terminology for subphenotypes, the need for further research into the potential co-existence or overlap of subphenotypes, which could affect treatment response, the investigation of interactions among clinical, biological, and physiological factors, the refinement of preclinical models, the identification of biomarkers from organs beyond the bloodstream, and the determination of outcome variables beyond the commonly used 28- or 90-day mortality endpoints [25].

In the present study, we aimed to describe the main pathophysiological contributors to ARDS. Moreover, we discuss the distinct subphenotypes of the disease and how they can influence potential therapeutic strategies, such as mechanical ventilation (MV) and fluid management.

## 2. Methods

A comprehensive literature search was performed using PubMed to identify relevant studies on fluid management in patients with TBI. The search terms included “*acute respiratory distress syndrome*”, “*lung injury*”, “*pathophysiology*”, “*mechanical ventilation*”, “*ventilator induced lung injury*”, “*subphenotypes*”, “*personalized therapy*”, “*tidal volume*”, “*positive end-expiratory pressure*”, “*biomarkers*”, “*fluid management*”, “*fluid resuscitation*”, “*fluid responsiveness*”, “*haemodynamic alterations*”, “*statins*”, and “*critical care*”. Boolean operators (AND, OR) and truncations were employed to refine and optimize the results. The search focused on articles published in English from the past 20 years (2005–2025), although key foundational studies published earlier were included to provide historical context and foundational knowledge. Additionally, a manual screening of the references from the selected studies was conducted to uncover further relevant literature. We included randomized controlled trials, as well as observational studies, that reported data on the pathophysiology and subphenotypes of severe respiratory failure and ARDS. Both peer-reviewed publications and preprints were considered eligible, whereas case reports and case series with fewer than five patients were excluded.

## 3. Pathophysiology of ARDS

### 3.1. Alveolo-Capillary Barrier Dysfunction

**Figure 1:**
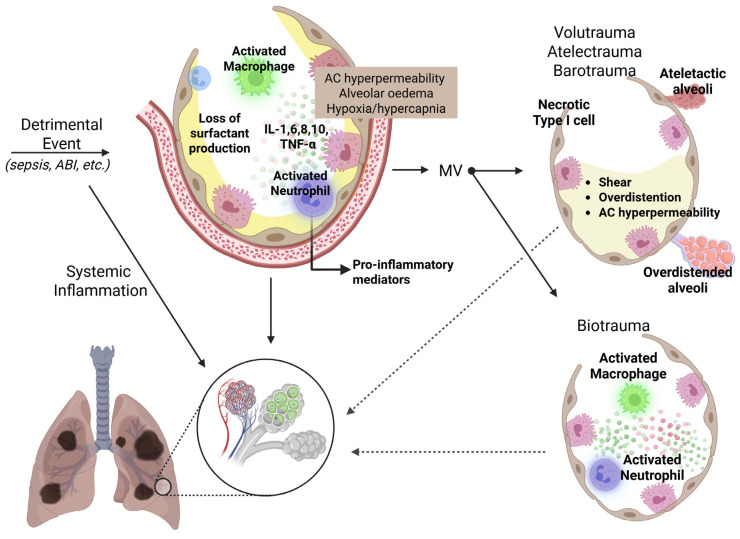
In patients with ARDS, the initial harmful insult triggers the release of inflammatory mediators, leading to the migration of activated immune cells, such as neutrophils, into the alveoli and pulmonary microcirculation. This exacerbates the pulmonary inflammatory response, further damaging the vascular endothelium and alveolar epithelium, increasing pulmonary capillary permeability, and causing the accumulation of protein-rich fluid in the alveoli. While mechanical ventilation is a cornerstone of ARDS management, it can also contribute to lung injury by inducing excessive alveolar distension (volutrauma), repetitive cyclic opening and closure of alveoli (atelectrauma), and a complex inflammatory cascade that drives both local and systemic inflammation (biotrauma). ABI: acute brain injury; ARDS: acute respiratory distress syndrome; IL: interleukin; MV: mechanical ventilation; TNF: tumor necrosis factor.

ARDS manifests clinically with dyspnea, refractory hypoxemia, reduced lung compliance, and diffuse alveolar infiltrates on chest imaging, characteristics that cannot be solely attributed to volume overload, cardiogenic pulmonary edema, or chronic lung disease [1,26,27,28]. Unlike pulmonary edema, which occurs due to the rapid filtration of fluid from the circulation into the lung’s extravascular spaces, exceeding its removal rate, pulmonary edema in ARDS primarily arises from an impairment in alveolo-capillary permeability rather than from hydrostatic pressure [29]. Indeed, in ARDS, injury to the alveolar epithelial and capillary endothelial cells of the alveolar–capillary barrier is typical, ranging from epithelial activation with adhesion molecule expression and activation of proinflammatory and procoagulant pathways to paracellular hyperpermeability due to damage to intercellular junctions, and even to epithelial cell necrosis with disruption of the alveolar basement membrane, leading to the accumulation of fluid, proteins, neutrophils, and red blood cells in the alveolar space (Figure 1) [11,30,31,32]. Moreover, the failure of normal transport mechanisms for alveolar epithelial fluid, which typically manages edema by transporting it into the interstitium for reabsorption into the circulation and eventual clearance via the lymphatic system, leads to exacerbated alveolar flooding [33]. In addition, the activated endothelium triggers the recruitment of neutrophils, which subsequently release their nuclear contents to form neutrophil extracellular traps in conjunction with activated platelets [34]. The increased permeability of the alveolar–capillary barrier is a key factor in the pathophysiology and management of ARDS, as even slight elevations in pulmonary hydrostatic pressure can greatly enhance the risk of pulmonary edema. At the same time, the need for positive-pressure ventilation underscores the importance of maintaining adequate cardiac preload, for example, in sepsis, which frequently causes vasodilatory shock and reduced cardiac output (CO), making early fluid resuscitation a critical aspect of ARDS management [35]. Furthermore, elevated pulmonary hydrostatic pressure can also intensify local inflammation and worsen hyperpermeability, likely as a result of the mechanical stretching of pulmonary vascular endothelial cells [36]. Moreover, microvascular thrombosis resulting from the activation of coagulation pathways exacerbates dead-space ventilation and worsens gas-exchange impairments in ARDS, thereby correlating with increased mortality rates [37]. Microbial pathogens, hyperoxia, aspiration of gastric contents, or mechanical forces in mechanically ventilated patients can directly cause epithelial damage, which can also arise from circulating factors like DAMPs, bacterial products, and toxins, activated adaptive and innate immunity, and inflammatory cascades [11,30,38,39,40,41,42].

Effective epithelial repair is a crucial process for edema resolution, restoration of barrier integrity, gas-exchange function, and prognosis in ARDS, and is mainly mediated by alveolar type (AT) II cells, which are less vulnerable to injury. Epithelial repair involves the proliferation of AT II cells to replace cell loss, followed by their subsequent transdifferentiation into AT I cells, whose function is critical to restore normal lung function, gas exchange, and barrier integrity. Although the exact molecular signaling pathways are not fully understood, accumulating research indicates that hypoxia-inducible factor 1α (HIF1α), Wnt/β-catenin, forkhead box protein M1 (FoxM1), keratinocyte growth factor (KGF), and hepatocyte growth factor (HGF), among others, promote AT II cell proliferation [43,44].

### 3.2. Dysregulated Pulmonary Inflammation

The clinical and pathological significance of ARDS is underscored by the accumulation of white blood cells, especially neutrophils, in the lung and alveolar spaces [17,45,46]. Neutrophils, typically absent in healthy airspaces, migrate from the lung vasculature into the airspaces during the early stages of ARDS, primarily mediated by resident tissue macrophages and recruited macrophages at the site, releasing harmful mediators such as reactive oxygen species, proteases, and proinflammatory lipid-derived substances like prostaglandins and leukotrienes [47,48]. Additionally, neutrophil extracellular traps [49], extracellular histones, and granular proteins, such as neutrophil elastase and myeloperoxidase released from dying neutrophils, act as DAMPs, causing epithelial and endothelial cell death, while circulating histones promote platelet aggregation, enhance neutrophil recruitment, and intensify inflammation [50,51]. Moreover, neutrophils, pivotal in both intravascular and extravascular compartments during LI, often collaborate with platelets, highlighting complex thrombo-inflammatory activities, which are implicated in disrupting alveolar–capillary and epithelial barriers in ARDS patients and experimental models [11]. In addition, inflammatory macrophages, entering the lung mainly through the capillary wall, induce cell death through various mechanisms, including the release of the TNF-related apoptosis-inducing ligand. Alveolar macrophages simultaneously regulate inflammation and influence epithelial permeability [47,48,52,53]. Recent research indicates that intra-alveolar inflammation in ARDS is regulated not only by macrophages but also by other immune cells, including lymphocyte subsets and dendritic cells, along with cytokine-mediated reactions [54,55]. Indeed, widespread lung inflammation characterizes ARDS, resulting in endothelial and epithelial damage and a subsequent rise in vascular permeability [56,57].

ARDS extends beyond the lungs, involving complex interactions between the pulmonary and other organ systems, and presents as a systemic inflammatory condition. Elevated levels of inflammatory cytokines, such as interleukin IL-1β, TNF-α, IL-6, and IL-8, are prominently found in both the bronchoalveolar lavage fluid (BALF) and the plasma of ARDS patients (Figure 1) [58], significantly contributing to tissue damage in critical organs and ultimately resulting in MODS [59].

### 3.3. Mechanical Stimuli in ARDS

MV remains a key element in managing ARDS. However, recognizing that it can also lead to LI represents a major breakthrough in ARDS research [12,60,61]. Due to the diverse nature of LI across different areas and the varying impacts of MV on the lung, it is likely that multiple mechanisms of ventilator-induced lung injury (VILI) occur concurrently within the lung [62,63], including exposure to elevated inflation transpulmonary pressures (barotrauma), excessive alveolar distension (volutrauma), and repetitive cyclic opening and closure of alveoli (atelectrauma). Additionally, apart from directly causing structural changes, these mechanical forces can initiate a complex inflammatory reaction, creating an inflammatory milieu in both localized lung tissues and throughout the body (biotrauma) (Figure 1) [18,64]. The risk of VILI is influenced by the interaction of mechanical factors, such as exceeding safe inspiratory pressure thresholds, applying harmful stretching forces rapidly enough to bypass natural adaptive responses, and sustaining a damaging pattern of high-pressure cycles over an extended period [65,66]. Indeed, VILI can be influenced by several static factors, including peak pressures, plateau and driving pressures, the level of positive end-expiratory pressure (PEEP), and the tidal volume (TV) delivered. Additionally, dynamic factors such as the rate of respiration, the amplitude of airflow, and the ratio of inspiratory time to the total respiratory cycle also play a significant role in its occurrence [12,67]. Currently, the ratio of TV to respiratory system compliance, known as the driving pressure during the passive inflation cycle, has emerged as the foremost ventilatory determinant linked to adverse outcomes [68,69]. Indeed, due to reduced lung compliance and uneven alveolar consolidation, ARDS causes volutrauma and barotrauma, hindering effective alveolar ventilation in dependent lung regions. Interestingly, during forceful spontaneous inhalation efforts, transpulmonary pressures may also increase as a result of markedly negative pleural pressures [70,71]. Additionally, these mechanical forces not only induce direct structural changes but also initiate a complex inflammatory response, leading to both localized and widespread inflammation, known as biotrauma [64]. Moreover, it has been demonstrated that MV increases alveolar–vascular permeability, facilitating the spread of inflammation from the lungs to other organs, potentially leading to multiple organ failure (MOF) and higher mortality. This MV-induced inflammatory response, known as biotrauma, arises from direct cellular trauma that disrupts cell walls and releases cytokines locally in the alveoli and into the bloodstream, as well as from mechanotransduction, a process where cells sense mechanical forces and convert them into cellular signaling events [61,72,73,74,75]. While the best ventilation strategy for each patient is still debated, the current recommendations include a protective ventilator strategy, generally avoiding lung overdistension and reducing cyclic atelectasis by appropriately using PEEP [12,61,76,77].

## 4. Subphenotypes in ARDS

Despite extensive preclinical research on the pharmacological treatments for ARDS, which has demonstrated encouraging results, the in-hospital mortality remains high, reaching approximately 40%, partially due to the heterogeneous nature of the disease [2,23]. Subsequently, it has been proposed that ARDS be categorized based on clinical parameters or by direct vs. indirect type of LI [21]. The term “subphenotype”, which includes prognostic and predictive elements, refers to a subgroup within a disease that exhibits elevated risk for worse outcomes or shares equivalent underlying biological factors and/or a different response to therapeutic strategies [78,79].

### 4.1. Biological Subphenotypes

**Figure 2:**
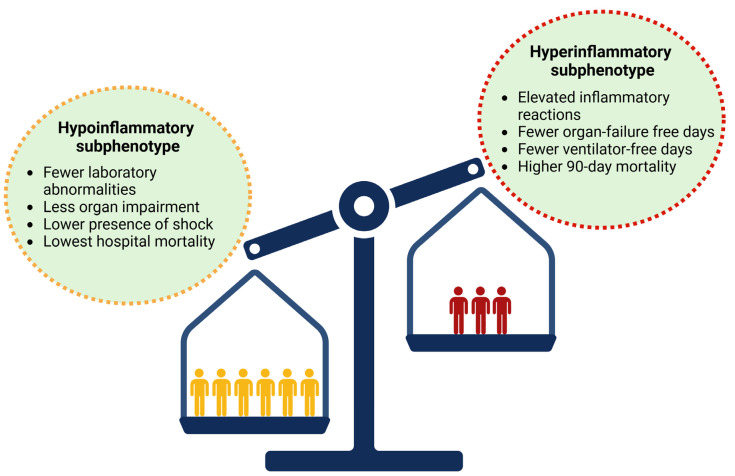
The hypoinflammatory and hyperinflammatory subphenotypes of ARDS exhibit distinct clinical characteristics, laboratory parameters, and outcomes, highlighting their relevance in disease progression and therapeutic response.

**Figure 3:**
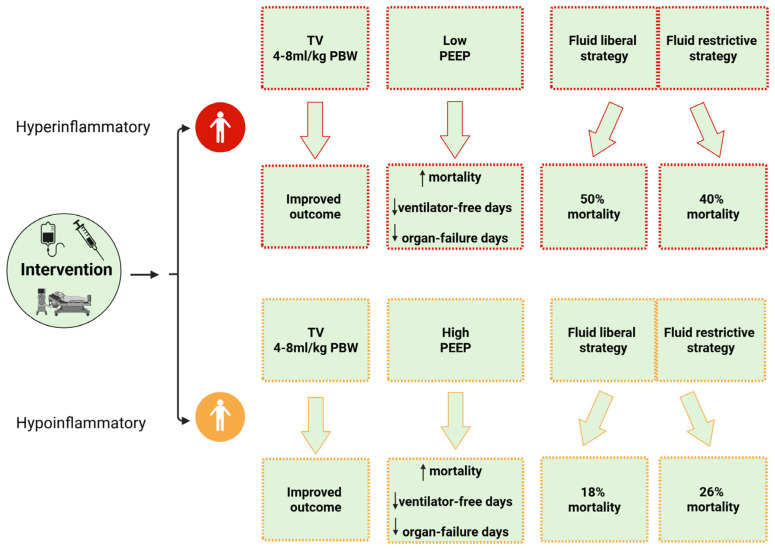
Distinct ARDS subphenotypes exhibit varying responses to treatment strategies, affecting clinical outcomes and mortality, thereby emphasizing the importance of personalized approaches to interventions such as ventilation strategies and fluid management. PEEP: positive end expiratory pressure; TV: tidal volume.

In the study of Calfee and co-workers (2014) analyzing data from the ARMA trial (Ventilation with Lower Tidal Volumes as Compared with Traditional Tidal Volumes for Acute Lung Injury and Acute Respiratory Distress Syndrome) [65], one-third of patients experience the hyperinflammatory subphenotype, with the primary inflammatory biomarkers identified including soluble tumor necrosis factor receptor 1 (sTNFR1), IL-6 and -8, von Willebrand factor (vWF), plasminogen activator inhibitor-1 (PAI-1), and intercellular adhesion molecule-1 (ICAM-1). Moreover, although the severity of ARDS and the impact of extrapulmonary organ failure were similar in both groups, clinical and ventilator parameters, such as systolic blood pressure, heart rate, minute ventilation, level of PEEP, and vasoactive support, could differentiate the two subphenotypes [21]. In addition, it has been highlighted that these subphenotypes, recognized within the first 36 h following the diagnosis of ARDS, exhibit a different response to several therapeutic measures, such as fluid management and the application of PEEP, which could explain the differences in 90-day mortality and organ failure-free and and ventilator-free days (Figure 2 and Figure 3) [21,80,81]. These findings are further supported by the study of Famous et al. (2017) [81], which reported the presence of the hyperinflammatory subphenotype in nearly one-third of 1000 retrospectively analyzed patients, with findings similar to those of Calfee et al. regarding clinical parameters, discriminating biomarkers, and mortality. In this study, trauma, pneumonia, and aspiration were predominantly related to the hypoinflammatory subphenotype, while sepsis was associated with the hyperinflammatory state [81]. These findings are consistent with the latent class analysis (LCA) of the HARP-2 (Inhibition with Simvastatin in Acute Lung Injury to Reduce Pulmonary Dysfunction-2) trial, which identified two distinct subphenotypes: class 1 (hypoinflammatory) and class 2 (hyperinflammatory). In this analysis, indirect causes of ARDS were significantly more common in the hyperinflammatory subphenotype. Moreover, patients with this subphenotype had significantly fewer ventilator-free days, fewer non-pulmonary organ failure-free days, increased vasopressor use, and higher 28-day and 90-day mortality compared to those with the hypoinflammatory subphenotype. In addition, patients with the hyperinflammatory phenotype exhibited higher concentrations of inflammatory mediators, such as soluble TNF receptor-1 (sTNFr-1) and IL-6, higher bilirubin and creatinine levels, and lower platelet counts [80]. Moreover, these observations are further supported by an LCA of the SAILS (Statins for Acutely Injured Lungs from Sepsis) trial, which also identified similar distinct phenotypes [82,83]. These subphenotypes were further confirmed through an LCA involving over 4000 patients from five randomized clinical trials (RCTs) and two observational cohorts (Figure 2) [21,80,81,84]. The aforementioned findings are consistent with those from a secondary analysis of the ROSE (Reevaluation of Systemic Early Neuromuscular Blockade) trial, which, however, identified a higher proportion of patients with the hyperinflammatory subphenotype, along with differences in mortality across subphenotypes. Distinct transcriptional profiles at baseline and Day 2 were observed for each subphenotype: the hyperinflammatory type showed gene expression patterns associated with innate immune activation and metabolic dysregulation, while the hypoinflammatory type was linked to adaptive immune responses and interferon (IFN) signaling [85]. Recently, a secondary analysis of a prospective observational cohort study, including 86 patients with the hyperinflammatory subphenotype and 1397 patients with the hypoinflammatory phenotype, reported stronger coagulation abnormalities, endothelial cell activation, inflammatory cascades involved in the pathogenesis of acute illness, and increased 30-day and one-year mortality [86]. Moreover, a meta-analysis by Jabaudon et al. (2018) highlighted that higher baseline plasma levels of the soluble receptor for advanced glycation end-products (sRAGE) predicted 90-day mortality in critically ill patients with ARDS, independently of ventilator parameters [87].

Stratifying ARDS patients based on pathobiological subtypes using specific biomarkers—indicative of mortality risk or response to targeted therapies—could help identify more homogeneous patient populations with distinct characteristics, thereby optimizing enrollment in clinical trials for those most likely to benefit from specific therapeutic interventions [21,88]. Indeed, biomarkers could play a fundamental role in enabling a personalized approach for patients with ARDS. However, despite the discovery of numerous biomarkers each year, only approximately 0.1% are currently recognized as reliable and routinely used clinical indicators [88,89]. The challenges related to the validation and utility of biomarkers in ARDS are multifactorial and are primarily associated with the heterogeneity of the syndrome, making the existence of a single, isolated biomarker unlikely [90]. Moreover, coexisting conditions that cause ARDS—or concurrent processes such as sepsis, pneumonia, aspiration, and trauma—may influence the biomarker expression currently under investigation [91,92]. In addition, study designs are considerably variable regarding patients’ characteristics, the timing of sample collection, which is critical in ARDS given the different phases of ARDS from the exudative to proliferative and subsequent to the fibrotic, the organ of sample collection, and finally, the analytical methods used among studies [89,90,93], influencing the validation of potential biomarkers. Furthermore, the heterogeneity among existing studies, which often introduces bias, combined with the retrospective nature of most research on ARDS subphenotypes, could limit the reproducibility of the results. Overcoming the aforementioned challenges could lead to the identification of accurate and reliable predictive markers in ARDS, establishing biological markers as significant tools for evaluating prognosis and guiding therapeutic interventions [94].

### 4.2. Subphenotypes by Etiology and Clinical Parameters

In addition to biological markers, the categorization of ARDS into subphenotypes has also been made using the clinical parameters (e.g., underlying cause, direct vs. indirect, timing of ARDS onset) and the physiological parameters (e.g., arterial partial pressure of oxygen/fraction of inspired oxygen (PaO_2_/FiO_2_) ratio, respiratory system compliance, elastance, shunt, and lung weight) [95,96,97,98,99].

The causes of ARDS can be categorized as pulmonary, such as pneumonia, which directly affects the alveolar epithelium, or extrapulmonary, such as sepsis and pancreatitis, which indirectly damage the lungs by causing endothelial injury [29,100]. It has been highlighted that patients with direct ARDS exhibit more severe pulmonary epithelial damage, milder endothelial injury, lower levels of inflammatory cytokines and vWF antigen (as plasma markers of endothelial injury), and lower overall illness severity and fewer organ failures [101,102].

Although patients with severe traumatic injuries are at higher risk of developing ARDS, due to factors such as hemorrhagic shock, massive transfusion of blood products, fluid resuscitation, and MV, trauma-associated ARDS is correlated with better outcomes and lower mortality compared to non-trauma-related ARDS [103,104,105,106]. Similarly, non-sepsis-associated ARDS is characterized by lower mortality and disease severity compared to septic ARDS patients [107,108]. Moreover, a retrospective study analyzing clinical data from 3875 patients included in three RCTs (ALVEOLI, FACTT, and SAILS) in patients with ARDS identified three distinct clinical phenotypes. Phenotype I was observed in 40% of the cohort and was characterized by fewer laboratory disturbances, less organ impairment, and the lowest in-hospital mortality rate (8%) compared with the other two phenotypes. Phenotype II was marked by elevated inflammatory reactions, the presence of shock, and a higher mortality rate (18%). Phenotype III was strongly associated with renal failure and acidosis and had the highest mortality rate (22%) [82,109,110,111]. This aligns with previous studies indicating that the development of acute kidney injury (AKI) in patients with ARDS is associated with a significantly increased risk of mortality [112,113].

During the Coronavirus Disease 2019 (COVID-19) pandemic, an LCA of the STOP-COVID trial (Study of the Treatment and Outcomes in Critically ill Patients with COVID-19) identified four subphenotypes of severe acute respiratory syndrome coronavirus 2 (SARS-CoV-2) infection based on clinical criteria. Subphenotype 1, which had the highest mortality (52.9%), was characterized by MOF, including AKI requiring renal replacement therapy, acidemia, and shock. Subphenotype 2 was associated with elevated C-reactive protein levels, the highest incidence of ARDS, and an early initiation of MV. Subphenotype 3 was marked by the greatest burden of chronic diseases, while subphenotype 4 exhibited mild physiological disturbances [114].

The timing of ARDS onset appears to impact outcomes, with late-onset ARDS being associated with higher mortality compared to early-onset cases [97,98]. Furthermore, patients with late-onset ARDS may experience a significantly more rapid decline following diagnosis [97,115]. In addition, patients with late-onset ARDS require longer durations of MV and experience prolonged stays in the ICU [97]. Importantly, a recent observational cohort study highlighted that patients with rapidly improving ARDS have better outcomes, lower plasma concentrations of inflammatory mediators, and are predominantly categorized as having the hypoinflammatory subphenotype [116].

### 4.3. Subphenotypes by Physiological Parameters

Regarding physiological parameters, large clinical trials in patients with moderate-to-severe disease, as defined by the PaO_2_/FiO_2_ criterion, have reported mortality rates in the control arms exceeding 40% [117,118,119]. Using a PaO_2_/FiO_2_ threshold of 150 mmHg, Maiolo et al. (2018) [120] classified patients with moderate ARDS to investigate potential homogeneity within the resulting subgroups and identified significant anatomical and physiological differences. Patients with more severe disease—defined by lower PaO_2_/FiO_2_ ratios—exhibited greater lung inhomogeneity, increased extravascular lung water, more collapsed lung areas, and higher peak pressures, PaCO_2_, and pH levels [120]. An LCA of a prospective cohort of 238 mechanically ventilated ARDS patients, using computer tomography (CT) findings and physiological variables such as dead space and lung inhomogeneity, revealed two distinct subphenotypes based on lung recruitability. The non-recruitable phenotype exhibited lower alveolar dead space and lung inhomogeneity, lower respiratory system elastance, and a smaller potentially recruitable lung volume. In contrast, the recruitable phenotype was characterized by lower respiratory system compliance, increased dead space, more severe disease, as defined by the PaO_2_/FiO_2_ ratio, and a higher risk of death [121]. These findings are in accordance with further research that highlighted an elevated dead space fraction as an independent risk factor for death [37]. Furthermore, driving pressure has been proven to be a prognostic marker in ARDS, with higher values being associated with increased mortality [68,122,123].

### 4.4. Subphenotypes by Radiographic Patterns

The radiologic presentation of ARDS is highly heterogeneous and includes a variety of morphological abnormalities such as bilateral pulmonary ground-glass opacities, alveolar consolidations, interstitial changes, or even normal-appearing lung parenchyma [29,124]. In addition, as ARDS progresses, the extent and distribution of radiographic abnormalities, such as shadowing and lesion range, can vary widely among patients, posing challenges for accurate diagnosis and effective treatment [125,126]. Studies assessing lung morphology using CT imaging have identified two distinct radiographic subphenotypes in ARDS. The first is a non-focal pattern characterized by widespread and irregular distribution of infiltrates, in which PEEP leads to significant alveolar recruitment. The second is a focal pattern, typically presenting with dorsal–inferior opacities, which tends to respond better to prone positioning [127,128].

Differences in radiographic patterns have also been observed when categorizing patients into pulmonary and extrapulmonary ARDS. Pulmonary ARDS typically presents with an asymmetric combination of consolidations and ground-glass opacities on CT imaging, whereas extrapulmonary ARDS more commonly shows a symmetric distribution of ground-glass opacities, as demonstrated by Goodman et al. (1999) [125]. However, in a study by Desai et al. (2001), aside from a significantly higher incidence of opacifications in nondependent lung regions among patients with pulmonary ARDS, no other differences between the pulmonary and extrapulmonary groups were observed [129]. Finally, certain radiological findings, such as intralobular and interlobular septal thickening, ground-glass opacities, traction bronchiectasis, and honeycombing, have prognostic value, with ground-glass opacities and honeycombing being positively related to fibroproliferation [130,131].

### 4.5. Cardiovascular Subphenotypes

Hemodynamic alterations and shock are common in ARDS and are associated with significant morbidity and mortality. The pathophysiology of hemodynamic instability in ARDS is multifactorial and involves mechanisms such as right ventricular dysfunction, pulmonary hypertension, the hemodynamic effects of MV on right ventricular function, and sepsis [132,133]. Nonetheless, the underlying mechanisms of circulatory failure in critically ill patients are highly heterogeneous, which can influence both prognosis and therapeutic management [134]. To better understand the pathophysiology of hemodynamic alterations and shock in ARDS patients and to explore the potential cardiovascular subphenotypes, Chotalia et al. (2023) [135] conducted a retrospective study using transthoracic echocardiography (TTE) findings and hemodynamic parameters in 801 ARDS patients, identifying four distinct circulatory failure phenotypes. Class 1 was the most frequent phenotype and was characterized by normal left and right ventricular function. Class 2, observed in approximately one-quarter of patients, was defined by a predominantly dilated right ventricle with preserved systolic function. Class 3, which was associated with the highest 90-day mortality, at nearly 80%, showed a dilated right ventricle with impaired systolic function. In contrast, Class 4 was characterized by high cardiac output and hyperdynamic left ventricular function [135]. An LCA by the same investigators in 305 patients with COVID-19 pneumonitis identified three distinct cardiovascular subphenotypes. Class 1, seen in slightly more than half of the patients, was characterized by normal right ventricular function. Class 2 showed right ventricular dilation with mostly preserved systolic function, while Class 3 exhibited right ventricular dilation with systolic impairment and was associated with the highest 90-day mortality, at 73% [136]. In a secondary analysis of the Fluid and Catheter Treatment Trial (FACTT), focusing on the pulmonary artery catheter arm, Siuba et al. (2025) [137] identified two distinct subphenotypes related to right ventricular–pulmonary arterial coupling. Subphenotype B was characterized by a higher heart rate, lower mean arterial pressure and arterial pH, and worse oxygenation, as indicated by the PaO_2_/FiO_2_ ratio. The tidal volumes did not differ significantly between groups, although patients in subphenotype B received higher levels of PEEP. Importantly, a liberal versus restrictive fluid strategy did not significantly affect mortality in either group [137]. Moreover, a meta-analysis of nine studies that included 1861 patients with ARDS, conducted by Sato et al. (2021), reports that right ventricular injury in ARDS is significantly associated with higher overall and short-term mortality [138].

## 5. Therapeutic Management Based on ARDS Subphenotypes

Retrospective studies have highlighted varying responses to therapeutic strategies, such as PEEP, prone positioning, fluid management strategies, and statin administration, among different subphenotypes of ARDS. However, pre-randomization studies are infrequent in clinical practice, primarily due to the scarcity of biomarker tests [20,21,24,80,81].

### 5.1. Ventilation Strategy

#### 5.1.1. Tidal Volume

Although it is well-established that a protective ventilatory strategy using low TV and PEEP improves mortality in patients with ARDS, it remains unclear whether this approach should be universally applied to all critically ill patients, as it may lead to self-inflicted LI and hypercapnia [12]. Moreover, when managing ARDS patients with low TV, careful attention should be given to physiological parameters, such as respiratory compliance, as these parameters help determine additional ARDS subphenotypes [96,139]. Indeed, the severity of ARDS may depend on respiratory compliance, as it mirrors the size of normally aerated lung volume or functional lung size [63,68]. A secondary analysis of the ARDSNet trial data demonstrated that ventilation with low TV can significantly improve mortality in patients whose respiratory system compliance is <0.6 mL/cmH_2_O/kg [140]. However, it has been suggested that ventilator parameters, including TV, should be individualized and titrated based on close monitoring of physiological indicators such as inspiratory capacity and driving pressure [141]. Technological advances, such as processors within ventilators and adaptive support ventilation, may help calculate the optimal TV while taking safety considerations into account [142]. Recently, studies have adopted a personalized approach to guide ventilation strategies in patients with ARDS, including TV adjustments based on lung physiology and morphology, underlying causes, radiographic findings, and biological phenotypes [141]. A multicenter RCT enrolled patients with moderate-to-severe early ARDS (LIVE trial) and assigned them to either a standard ventilation strategy or an individualized approach guided by lung morphology. The control group received conventional protective ventilation with a TV of 6 mL/kg predicted body weight. In contrast, the personalized strategy varied according to lung morphology: patients with focal ARDS received a TV of 8 mL/kg, lower PEEP, and were positioned prone, whereas those with non-focal ARDS were managed with a TV of 6 mL/kg, higher PEEP, and underwent recruitment maneuvers [24]. The personalization of ventilator strategy was not associated with reduced mortality, possibly due to the misclassification of 21% of patients as having focal or non-focal ARDS, which may have contributed to elevated 90-day mortality in this population [24]. However, when misclassified patients were excluded, the post hoc analysis demonstrated a mortality benefit in favor of a personalized MV strategy [20].

#### 5.1.2. Positive End-Expiratory Pressure

The application of higher PEEP is a cornerstone in managing ARDS, as it promotes alveolar recruitment and reduces opening/closing damage, lung stress, and intrapulmonary shunting [143]. However, although encouraging data suggest that higher PEEP is associated with improved outcomes in severe ARDS, the optimal method for determining the best PEEP based on oxygenation and respiratory mechanics remains uncertain. This uncertainty is likely due to the heterogeneous responses, including varying responses to PEEP, observed among patients with ARDS [21,144,145,146], largely attributed to the distinct subphenotypes identified in ARDS. Indeed, using data from the ALVEOLI trial, Calfee et al. (2014) [21] highlighted that the effects of PEEP on mortality differ significantly between the two observed subphenotypes (hypoinflammatory and hyperinflammatory). Specifically, patients with the hypoinflammatory subphenotype had higher mortality and lower ventilator-free and organ failure-free days when randomized to the high PEEP strategy compared to those who received low PEEP (Figure 3). In contrast, patients with the hyperinflammatory subphenotype exhibited higher mortality and lower ventilator-free and organ failure-free days with the low PEEP strategy compared to high PEEP [21]. A recent study analyzing data from 1559 patients with ARDS enrolled in the previously published Higher vs. Lower Positive End-Expiratory Pressures in Patients With ARDS (ALVEOLI) trial [147] and the Alveolar Recruitment in ARDS Trial (ART) [109] found that, in patients with less severe disease and less pronounced inflammation, the application of higher PEEP was associated with a higher 28-day probability of death [148]. This can be attributed to the harmful effects of the high PEEP strategy, including overdistension of well-aerated lung areas, hemodynamic compromise, and impairment of gas exchange, which may potentially lead to extrapulmonary organ failure in patients with less-severe inflammation [148,149]. Recently, the ESICM guidelines on ARDS, which updated the 2017 guidelines, could not provide a recommendation for or against routine PEEP titration to improve mortality in ARDS patients, particularly regarding a higher PEEP/FiO_2_ strategy versus a lower PEEP/FiO_2_ strategy [150]. Nonetheless, the current official American Thoracic Society clinical practice guidelines for managing ARDS recommend a higher PEEP strategy without lung recruitment maneuvers, compared to lower PEEP, in patients with moderate-to-severe ARDS and a TV of 4–8 mL/kg predicted body weight (PBW) (Figure 3) [149].

### 5.2. Fluid Management

Fluid management in ARDS remains a significant challenge, as it has been shown that fluid overload negatively affects prognosis, with early positive fluid balance being associated with prolonged MV, longer ICU stay, and increased mortality [110,151]. Otherwise, a conservative fluid strategy could result in detrimental effects, particularly if hypoperfusion co-exists, including the development of acute renal failure [152] and long-term cognitive dysfunction [153]. Although the FACTT trial failed to demonstrate an overall benefit for 60-day mortality, the restrictive fluid strategy was associated with improved lung function and a shorter duration of MV without an increase in extrapulmonary organ failures, supporting the use of conservative fluid management in patients with LI [110]. However, it has been convincingly demonstrated that mortality outcomes vary significantly between distinct subphenotypes, with heterogeneous responses to fluid strategies, highlighting the need for individualized treatment approaches. Indeed, Famous et al. (2017) [81] demonstrated that 90-day mortality differed significantly between fluid management strategies in the hyperinflammatory subphenotype compared to the hypoinflammatory subphenotype. Specifically, mortality in the hypoinflammatory subphenotype was 18% with a fluid-liberal strategy versus 26% with a fluid-restrictive strategy, while mortality in the hyperinflammatory subphenotype was 50% with a fluid-liberal strategy and 40% with a fluid-restrictive strategy (Figure 3) [81]. As mentioned above, a secondary analysis of the FACTT trial identified three distinct phenotypes of ARDS based on clinical criteria: clinical phenotypes I, II, and III. The authors also emphasized the significant impact of fluid management strategies on 60-day mortality among the different phenotypes. In particular, patients with phenotype II demonstrated an overall benefit in 60-day mortality when a conservative fluid management strategy was employed. In contrast, phenotype III patients experienced higher 60-day mortality under the same strategy, highlighting the complexity of fluid management in older patients with renal failure [111].

### 5.3. Statins

The anti-inflammatory and immunomodulatory properties of statins, including their ability to reduce the synthesis of cytokines and chemokines, have generated increasing interest in their potential use for managing ARDS [154,155]. However, RCTs and a meta-analysis have not demonstrated a significant clinical benefit [156,157]. Nevertheless, an RCT in patients with severe sepsis showed that prior statin users had lower baseline IL-6 concentrations and improved outcomes [158], suggesting that statins may be beneficial in managing specific ARDS subpopulations, such as certain subphenotypes. Indeed, a secondary analysis of the HARP-2 trial, which enrolled 539 patients, aimed to identify subphenotypes with different responses to therapeutic interventions. It found that patients with the hyperinflammatory subphenotype receiving simvastatin had improved survival at both 28 and 90 days compared to those receiving placebo [80]. This effect is likely associated with the anti-inflammatory and immunomodulatory properties of simvastatin [159]. In contrast, a secondary analysis of the SAILS trial did not demonstrate a beneficial effect of rosuvastatin in patients with the hyperinflammatory subphenotype [83]. Potential hypotheses to explain the discrepancy between the findings of the HARP-2 trial and the SAILS study, as suggested by the SAILS investigators, include the hydrophilic or lipophilic nature of the statin, as it is well established that the plasma protein expression of inflammatory biomarkers depends on this property. Additionally, despite the stronger lipid-lowering potency and better safety profile of rosuvastatin, its anti-inflammatory and immunomodulatory effects may differ significantly from those of simvastatin [83]. Indeed, as previously mentioned, the pathophysiology of ARDS is closely linked to the release of inflammatory mediators, including IL-1β, IL-6, TNF-α, IL-8, IL-18, monocyte chemoattractant protein-1 (MCP-1), and IFN-γ [160,161,162,163,164,165,166,167]. It has been well-demonstrated in both clinical and experimental research that simvastatin can inhibit the expression of a variety of the aforementioned inflammatory mediators, including IL-6, IL-8, MCP-1, IL-17, and TNF-α. In contrast, the anti-inflammatory profile of rosuvastatin includes reductions in the expressions of the interleukin 18 receptor accessory protein (IL18RAP), the high-sensitivity C-reactive protein (hs-CRP), TNF-α, matrix metalloproteinase-9 (MMP-9), MCP-1, and TNF-α [168,169,170,171,172,173]. Further research is emerging to investigate the potential role of various statins in the management of ARDS.

## 6. Future Directions

A precise differentiation of ARDS subphenotypes based on biological, physiological, clinical, and other relevant criteria will enrich clinical trials, increase study effectiveness, and may improve the ability to identify effective treatments—ultimately helping to translate the potential of precision medicine into clinical practice [174,175]. However, classifying ARDS into subphenotypes presents several challenges, including variability in patient demographics, clinical settings, and sample collection, as well as difficulties in validation [81,176]. These issues highlight the need for standardized methods and consistent research approaches as critical elements in accurately defining subphenotypes. Moreover, the rapid identification of these patients at the bedside remains a critical challenge—one that could potentially be addressed through point-of-care biomarker assays [81,177]. However, although no single biomarker is absolutely accurate for identifying ARDS subphenotypes, existing biomarkers—or combinations of them—may still aid in their identification at the bedside. A retrospective analysis of data from three major ARDS Network randomized controlled trials, namely ARMA, ALVEOLI, and FACTT (totaling 2022 patients), along with the SAILS trial (715 patients), applied machine-learning techniques to identify key classifier variables. These variables were used to develop logistic regression models aimed at distinguishing ARDS subphenotypes. The model with the highest predictive accuracy was validated using the SAILS dataset, and its prognostic performance was further assessed in two external clinical trial cohorts, START and HARP-2 [178]. The study demonstrated that simple classification models using just three to four variables, such as IL-6, IL-8, sTNFR-1, protein C levels, vasopressor use, and bicarbonate therapy, can reliably differentiate ARDS subphenotypes. Nevertheless, given the retrospective nature of most studies investigating ARDS subphenotypes—and associated limitations such as missing data and potential biases—well-designed, biomarker-oriented prospective trials are needed. These should also assess biomarker levels at various stages of the disease to determine when specific therapies may be most beneficial [179].

Despite intensive research over recent decades, no effective therapy for ARDS has been established, and current therapeutic management remains mainly supportive [180]. Preclinical studies have demonstrated that mesenchymal stromal cells (MSCs) derived from sources such as bone marrow, umbilical cord, and adipose tissue possess therapeutic potential to mitigate lung inflammation and fibrosis, showing encouraging efficacy in the treatment of ARDS [180,181]. MSCs exert multiple bioactivities, including immunomodulation and inhibition of inflammation through the modulation of neutrophil, macrophage, and T-cell function; regulation of immune homeostasis; stimulation of tissue repair; enhancement of sodium-dependent alveolar fluid clearance; and modulation of pulmonary vascular endothelial permeability [180,182,183,184]. Moreover, MSCs exhibit antimicrobial activity through the secretion of antimicrobial substances and the stimulation of monocyte/macrophage phagocytosis [180]. However, key unresolved issues in the use of MSCs for the treatment of ARDS include the optimal MSC source, dosage, single versus multiple administrations, and patient selection, as it is hypothesized that MSC therapy may be more effective in patients with the hyperinflammatory subphenotype [180,185].

Preclinical research in recent years highlights that anti-inflammatory therapeutic strategies may have beneficial effects in ARDS, particularly in specific subphenotypes [186]. Corticosteroids are the most extensively studied anti-inflammatory drugs in ARDS, though their use remains controversial. Several RCTs and meta-analyses suggest a mortality benefit and more ventilator-free days, particularly when treatment is initiated within 14 days of ARDS onset [177,187,188,189]. The significant role of corticosteroids in patients with severe respiratory failure became more evident during the COVID-19 outbreak, when the use of dexamethasone was shown to reduce mortality in both mechanically ventilated patients and those receiving oxygen without MV, as well as to increase ventilator-free days [190,191]. However, harmful effects have also been reported, especially in ARDS related to influenza infection [192]. These data raise the question of whether corticosteroid administration may confer a benefit or cause harm depending on the specific ARDS subphenotype. Indeed, a recent retrospective study investigating the role of glucocorticoids in patients with distinct ARDS subphenotypes found that, in patients with the class 2 subphenotype—characterized by more severe acidosis, impaired kidney function, elevated glucose levels, lower blood pressure, and more frequent use of vasoactive agents—glucocorticoid administration was associated with reduced 28-day survival [193]. Future research through well-designed trials is needed to clarify the potential benefits of corticosteroids in specific ARDS subphenotypes, the underlying causes of ARDS, and the optimal dosage and duration of therapy. Moreover, the potential benefits of other anti-inflammatory therapies, such as anti-IL-6 or anti-TNF agents, and novel substances, for example, interleukin-1 receptor-associated kinase 4 (IRAK4) inhibitors, should be further evaluated in the context of the syndrome’s heterogeneity [194,195,196].

## 7. Conclusions

The breakdown of endothelial and epithelial barriers, along with the activation of inflammatory cascades, plays a central role in the pathophysiology of ARDS. Despite advances in understanding the pathophysiology of the disease, effective therapies are limited and mainly supportive, probably due to the existence of distinct ARDS subphenotypes. Although substantial progress has been made in understanding the heterogeneity of ARDS, significant research gaps remain, including the identification of reliable biomarkers, the stability of subphenotypes, and the integration of precision medicine approaches, all of which are critical for developing effective therapeutic strategies and improving patient outcomes. Optimizing the identification and characterization of discrete ARDS subphenotypes based on clinical, biological, physiological, and radiographic criteria will deepen our understanding of ARDS pathophysiology and support the development of more personalized and precise therapeutic strategies.

## Data Availability

Not applicable.
